# Evidence of CD40L/CD40 pathway involvement in experimental transfusion-related acute lung injury

**DOI:** 10.1038/s41598-019-49040-0

**Published:** 2019-08-29

**Authors:** Sofiane Tariket, Hind Hamzeh-Cognasse, Sandrine Laradi, Charles-Antoine Arthaud, Marie-Ange Eyraud, Thomas Bourlet, Philippe Berthelot, Olivier Garraud, Fabrice Cognasse

**Affiliations:** 1 Université de Lyon, GIMAP-EA3064, Saint-Etienne, France; 2 Établissement Français du Sang Auvergne-Rhône-Alpes, Saint-Etienne, France; 3 Institut National de la Transfusion Sanguine, Paris, France

**Keywords:** Inflammation, Translational research

## Abstract

Platelet transfusions can cause adverse reactions in their recipients, including transfusion-related acute lung injury (TRALI). The pathophysiology of TRALI depends on a number of signaling pathways and the inflammatory role played by blood platelets remains controversial. Platelets are important in inflammation, particularly via the immunomodulator complex CD40/CD40L. We studied the specific function of the CD40/CD40L interaction in regulating an experimental TRALI Two-hit model. A mouse model of immune TRALI was triggered by injection of LPS and an anti-MHC I antibody, and the effect of injection of a neutralizing anti-CD40L antibody before induction of TRALI investigated. The characteristics of TRALI were decreased body temperature, pulmonary lesions, and immune cell infiltration into the alveolar space. Pulmonary infiltration was evaluated by blood counts of specific immune cells and their detection in lung sections. Inhibition of the CD40/CD40L immunomodulator interaction significantly reduced communication between immune and/or endothelial cells and the development of pulmonary edema. Hence, our results indicate that targeting of the CD40/CD40L interaction could be an important method to prevent TRALI. While considering that our work concerned a mouse model, we postulate that improvement of the conditions under which platelet concentrates are prepared/stored would assist in alleviating the risk of TRALI.

## Introduction

A “two-hit” model has been proposed for the classical form of transfusion related acute lung Injury (TRALI)^1^, whereby inflammatory conditions present in some transfused recipients strongly predispose them to the development of pathology, resulting in changes to the phenotypes of inflammatory cells, such as neutrophils, platelets, and endothelial cells^2,3^; this is the priming event. The second hit is assumed to result from transfusion of human leucocyte antigen (HLA) and human neutrophil antigen (HNA) antibodies^4^ and/or soluble mediators^5^ (sCD40L - soluble CD40 ligand, Bioactive lipids…) present in blood. A mouse model was proposed, in 2009, to mimic the hypothesis of the “two-hit” pathophysiology of human TRALI^6^. This experimental TRALI is based on a first injection of lipopolysaccharide (LPS), to reproduce the priming step, followed by an intravenous injection of anti-MHC I mAb, to induce pulmonary edema. CD40 is a membrane protein found on many immune and inflammatory cells, and CD40 ligand (CD40L) is also expressed on the surface of various immune cells^7^. Soluble CD40L (sCD40L) is an agonist of CD40, 95% of which is secreted by platelets^8^. The interaction between CD40 and CD40L is central to numerous innate and adaptive immune functions^9^, and to many pathologies^10^; the CD40/CD40L interaction is considered pro-inflammatory^11^ and sCD40L appears to impact transfusion reactions and to accumulate with time in storage, particularly in platelet concentrate (PC)^12–15^. The incidence of typical TRALI can be greatly reduced by the removal of plasma-rich blood components, which include anti-MHC class I or class II^16^. Moreover, the substitution of PC plasma with platelet additive solution also led to a marked reduction in inflammatory complications in general, and TRALI in particular^17^. Nevertheless, cases of pTRALI and TRALI continue to be reported, since this pathology appears to be affected not only by the transfusion product, but also by patient characteristics^18^. Several factors justify interest in sCD40L in the context of TRALI.

First, from a transfusion perspective, sCD40L levels correlate with PC storage time^19,20^. Blumberg *et al*. demonstrated that transfusion, febrile, and allergic reactions were significantly correlated with high levels of sCD40L in PC^21^. Nevertheless, the role of the CD40/CD40L interaction in TRALI is controversial. Some reports unequivocally indicate its involvement^22^ while others deny such claims^23^. Second, this molecular complex, which is pivotal in both innate and adaptive immunity^11^, is often implicated in other forms of Acute lung injury (ALI) such as sepsis^24–27^. Such pathology depends on: (i) the formation of a cellular tri-complexes of neutrophils, endothelial cells, and platelets; (ii) transmigration of neutrophils and platelets into the alveolar space; and (iii) pulmonary complications^28^. Further, significant levels of sCD40L are observed in TRALI-implicated PCs and in the plasma of 67% of TRALI patients post-reaction^22^. Finally, in three animal models of ALI, induced by hyperventilation^29^, radiation^30^, or following endotoxemia^31^, pathophysiology appeared to be CD40/CD40L dependent.

Given the severity of TRALI and the continued use of transfusions for severely affected patients, risk reduction strategies targeting key molecules, including serum sCD40L, represent a potential route to improvement of this condition. In this study, we investigated sCD40L as a therapeutic target in a mouse two-hit model of TRALI. We described the central role of CD40/CD40L in intercellular communication as well as the migration of polymorphonuclear cells into alveolar space, a key phenomenon for TRALI induction. We also showed that CD40/CD40L inhibition is effective in the prevention of pulmonary edema.

## Results

### Anti-CD40L monoclonal antibody prevented pulmonary edema in the TRALI mouse model

**Figure 1:**
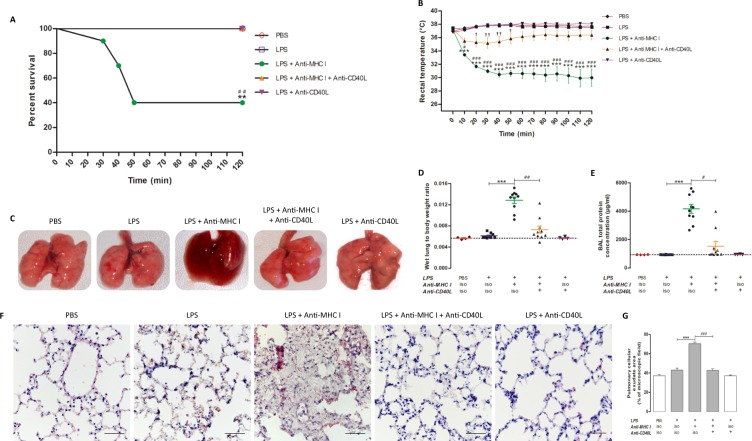
Evaluation of TRALI development. Survival curves (**A** - n = 4) and rectal temperatures (**B** - n = 10) were measured for each experimental group. The general appearance of the lungs (**C**) is represented for each group of mice. Wet lung to body weight ratio (**D**) and BAL total protein concentration (**E**) were measured for each group of mice. Lung microarchitecture is presented after H&E staining (**F**) for each group of mice (Original magnification x400). Scale bar, 50 μm. Pulmonary cellular exudate area was measured in ratio to the microscopic field. (**G**) Data are presented as means ± SEM. *p < 0.05, **p < 0.01, and ***p < 0.001 indicate differences between the [LPS] (n = 4) and [LPS + anti-MHC I] (n = 10) groups; ^†^p < 0.05, ^††^p < 0.01, and ^†††^p < 0^.^001 indicate differences between the [LPS] and [LPS + anti-MHC I + anti-CD40L] (n = 10) groups; and ^#^p < 0.05, ^##^p < 0.01, and ^###^p < 0.001 indicate differences between the [LPS + anti-MHC I] and [LPS + anti-MHC I + anti-CD40L] groups. (A) Except for condition LPS + antiMHC1, the values of the other conditions are confused for 100% of percent survival.

Injection of anti-MHC I (H2k^d^) mAb in Balb/c mice, conditioned by prior (24 h) LPS injection, resulted in mortality of 60% of animals within 2 h. BALB/c wild-type mice conditioned by prior (24 h) LPS injection alone had no effect on mortality. Treatment with anti-CD40L neutralizing antibody 30 min prior to anti-MHC I injection completely prevented mortality at 2 h (Fig. 1A). Mice in the [LPS + anti-MHC I] group, which mimicked TRALI, were hypothermic, with rectal temperatures approximately 8 °C below those of both [PBS] and [LPS] control group mice. Injection of anti-CD40L neutralizing antibody, 30 min before anti-MHC I antibody, maintained normothermia. The mean temperature reduction in [LPS + anti-MHC I + anti-CD40L] mice was ≤ −3 °C between 20–50 min, and in the last hour the difference was <−2 °C and not significantly different from controls (Fig. 1B). Pulmonary edema was more pronounced in the [LPS + anti-MHC I] group than in [LPS] control group. The injection of anti-MHC I after priming with LPS induced a dramatic deterioration of the lungs, as visualized by their violet color and moist appearance, suggestive of pulmonary infiltration (Fig. 1C). In contrast, the general appearance of the lungs in [LPS + anti-MHC I + anti-CD40L] mice was equivalent to that of [LPS] controls (Fig. 1C). Significant increases were observed in wet lung mass/body mass ratio and total protein in bronchoalveolar lavage (BAL) in [LPS + anti-MHC I] mice compared with [LPS] controls (p < 0.001), while treatment with intravenous anti-CD40L significantly reduced these two parameters (Fig. 1D,E). H&E staining of the pulmonary interstitium revealed pulmonary infiltration, evidenced by pulmonary cellular exudate, in [LPS + anti-MHC I] mice, while the pulmonary parenchyma of [LPS] controls and [LPS + anti-MHC I + anti-CD40L] treated mice showed no evidence of infiltration (Fig. 1F; quantified in Fig. 1G). These results suggest that prophylactic injection of anti-CD40L neutralizing antibodies could prevent pulmonary edema triggered by treatment with LPS and anti-MHC I and consequently the development of TRALI in this model.

### Anti-CD40L reduces lung infiltration by circulating leukocytes and platelets

**Figure 2:**
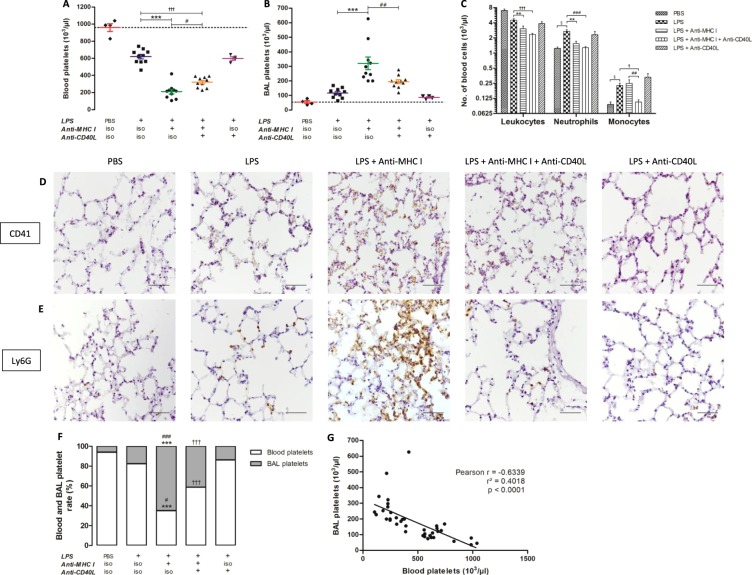
Evaluation of cell migration. Numbers of platelets in peripheral blood (**A**) and BAL (**B**), and of leukocytes, neutrophils, and monocytes in peripheral blood (**C**) were measured for each group of mice. Immunohistochemistry staining characteristic of platelet (CD41) (**D**) and neutrophil (Ly6G) (**E**) infiltration in the lungs are presented for each mouse group (Original magnification x400). Scale bar, 50 μm. Blood and BAL platelet proportions are presented for each group of mice. (**F**) Data are presented as means. ^§^p < 0.05, ^§§^p < 0.01, and ^§§§^p < 0.001 indicate differences between the [PBS] (n = 4) and [LPS] (n = 10) groups; *p < 0.05, **p < 0.01, and ***p < 0.001 indicate differences between the [LPS] and [LPS + anti-MHC I] (n = 10) groups; ^†^p < 0.05, ^††^p < 0.01, and ^†††^p < 0.001 indicate differences between the [LPS] and [LPS + anti-MHC I + anti-CD40L] (n = 10) groups; and ^#^p < 0.05, ^##^p < 0.01, and ^###^p < 0.001 indicate differences between the [LPS + anti-MHC I] and [LPS + anti-MHC I + anti-CD40L] groups. Correlation between blood and BAL platelet levels (**G**) was evaluated for all mice (n = 38). Spearman’s correlation and the coefficient of determination are represented by r and r², respectively, and p < 0.05 was considered statistically significant.

Levels of circulating platelets were slightly reduced after LPS injection, and subsequent anti-MHC I injection significantly exacerbated this thrombocytopenia (p < 0.001). Administration of anti-CD40L prior to anti-MHC I injection significantly limited the severity of the thrombocytopenia (p < 0.05), although platelet levels remained significantly lower compared with untreated controls (p < 0.001) (Fig. 2A). LPS injection alone produced a modest increase in platelets in BAL. After anti-MHC I lesional induction, the BAL platelet level increased by approximately 2.6 times on average as compared to the [LPS] control group. Prior Injection of anti-CD40L, significantly reduced the platelet infiltrate (p < 0.01) (Fig. 2B). Circulating levels of leukocytes, particularly neutrophils, increased significantly following LPS injection (p < 0.05), and subsequent treatment with anti-MHC I normalized the number of neutrophils. Moreover, injection of LPS + anti-CD40L (without anti-MHC I) did not alter baseline neutrophil levels observed in TRALI mice (LPS + anti-MHC I) treatment (Fig. 2C). LPS injection induced a significant elevation in monocytes (p < 0.05), which was maintained following injection of anti-MHC I antibodies (p < 0.01); however, levels decreased on injection with the anti-CD40L antibody, returning to basal levels (PBS control) (Fig. 2C).

In parallel, examination of the pulmonary parenchyma showed a greater infiltration of platelets and neutrophils in [LPS] mice than in the [PBS] control group. The addition of anti-MHC I amplified the infiltration whereas the injection of anti-CD40L [LPS + anti-MHC I + anti-CD40L] decreased and normalized levels of platelets (Fig. 2D) and neutrophils (Fig. 2E). Platelet proportion in BAL samples, considered partly migratory, were increased in [LPS + anti-MHC I] mice compared with [LPS] control and [LPS + anti-MHC I + anti-CD40L] treated groups, and were significantly higher in [LPS + anti-MHC I + anti-CD40L] group (41.34%) compared with [LPS] mice (17.74%) (p < 0.001) (Fig. 2F). Blood platelet numbers were significantly and negatively correlated with BAL platelet levels (p < 0.0001 and Pearson r = −0.6339) (Fig. 2G). Hence, injection of anti-CD40L antibody considerably affected platelet and leukocyte levels in the pulmonary parenchyma.

### Anti-CD40L antibody limits neutrophil and platelet activation induced by LPS and anti-MHC I antibody to a limited extent in blood and sustainably in lungs

**Figure 3:**
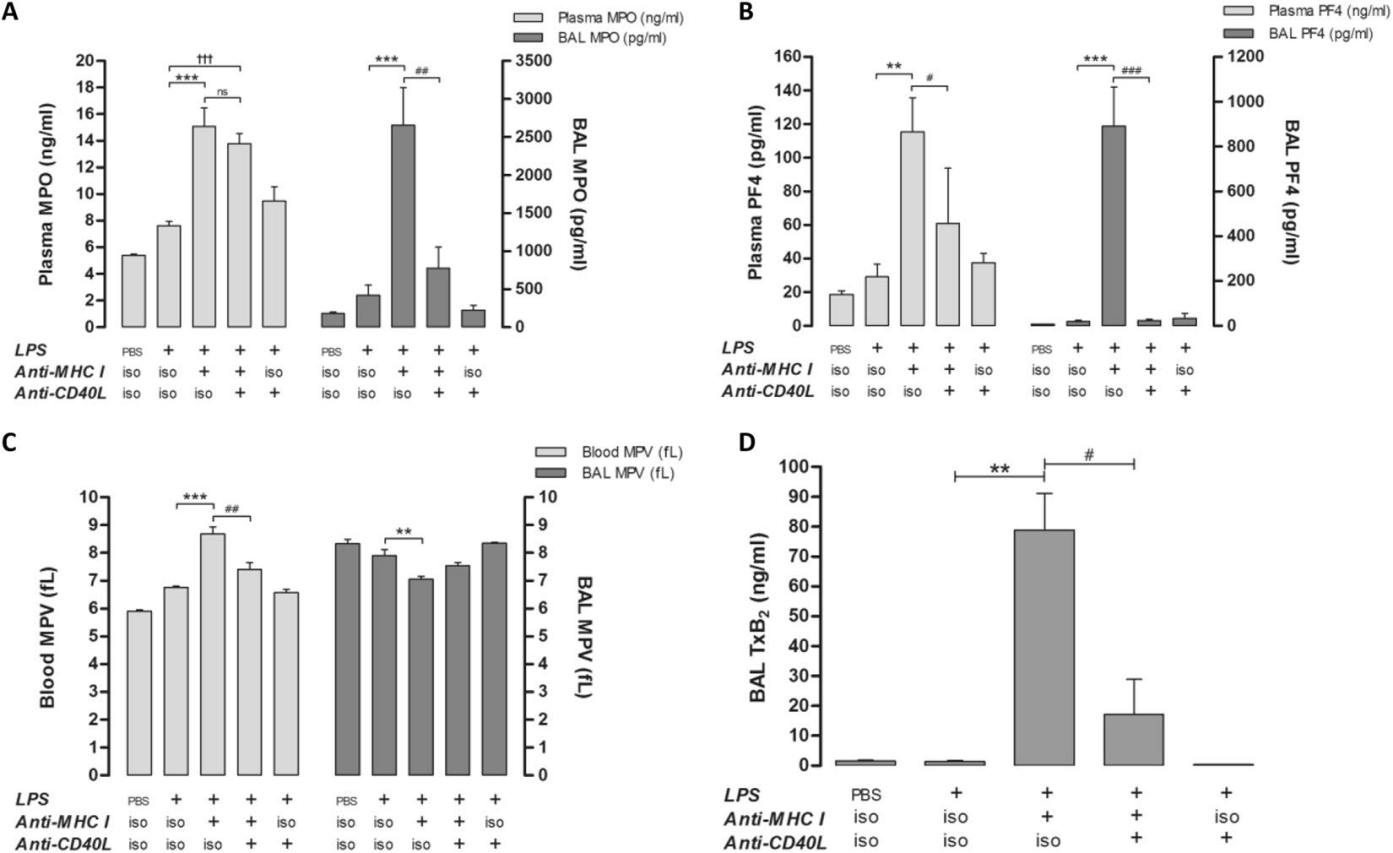
Evaluation of neutrophil and platelet activation. Quantification of MPO (**A** - n = 10) and PF4 (**B** - n = 10) in both plasma and BAL. MPV, in whole blood and BAL (**C** - n = 10), was determined by MS4® analysis for each group of mice. TxB_2_ in BAL (**D** - n = 10) was evaluated by ELISA for each group of mice. Data are presented as means ± SEM (n = 10). *p < 0.05, **p < 0.01, and ***p < 0.001 indicate differences between the [LPS] and [LPS + anti-MHC I] groups; ^†^p < 0.05, ^††^p < 0.01, and ^†††^p < 0^.^001 indicate differences between the [LPS] and [LPS + anti-MHC I + anti-CD40L] groups; and ^#^p < 0.05, ^##^p < 0.01, and ^###^p < 0.001 indicate differences between the [LPS + anti-MHC I] and [LPS + anti-MHC I + anti-CD40L] groups.

The levels of activated neutrophils and platelets were evaluated by measuring myeloperoxidase (MPO) and platelet factor-4 (PF4), respectively. Concentrations of MPO (Fig. 3A) and PF4 (Fig. 3B) were considerably increased in [LPS + anti-MHC I] mice compared with [LPS] controls in both plasma and BAL. Anti-CD40L reduced levels of PF4 in the blood and of FP4 and MPO in BAL, 1.9-, 33-, and 3.5-fold, respectively (Fig. 3A,B). Mean platelet volume (MPV), a characteristic of platelet activation^32^, was evaluated to assess the pathophysiological involvement of platelets in TRALI. An increase in MPV in [LPS + anti-MHC I] versus [LPS] mice was observed in blood, but not BAL, where basal MPV was high. Anti-CD40L antibodies neutralized MPV increases in the blood, with levels equivalent to those observed after treatment with LPS alone; however, anti-CD40L antibody treatment also led to a further increase in MPV in the lungs compared with [LPS + anti-MHC I] mice (Fig. 3C). Generally, platelet MPV in BAL was negatively correlated with BAL platelet count (Fig. S2). To confirm BAL platelet activation in [LPS + anti-MHC I] mice, the concentration of thromboxane B_2_ (TxB_2_) was measured. The levels of TxB_2_ in [LPS + anti-MHC I] mice were elevated compared with both control [LPS] and [LPS + anti-MHC I + anti-CD40L] mice (Fig. 3D), consistent with the results of PF4 assessment (Fig. 3B). TxB_2_ was only measured in BAL, because the sampling method precluded its measurement in blood. Hence, our results indicate that platelet activation is reduced by anti-CD40L, in both blood and lungs. In contrast, neutrophil activation was apparently irreversible in the blood, despite the inhibition of the CD40/CD40L immune complex (sCD40L).

We perform additional experiments to examine whether the interaction between human platelets and human neutrophils is responsible for amplified platelet sCD40L production. We observed a significant increase of activated phenotype (% CD41+/CD62P+ back gating on CD41+/CD15+) concerning Platelets before neutrophils binding compared to Platelets after neutrophils binding, respectively 3.83+/−0.99 *vs* 43.53+/−6,64. We investigated the sCD40L (ng/mL) release in the same condition and observed no significant modulation, respectively 0.05+/−0.01 *vs* 0.07+/−0.01.

### Effect of anti-CD40L on levels of NPA in blood and lung: importance of Macrophage-1 antigen (Mac-1)

**Figure 4:**
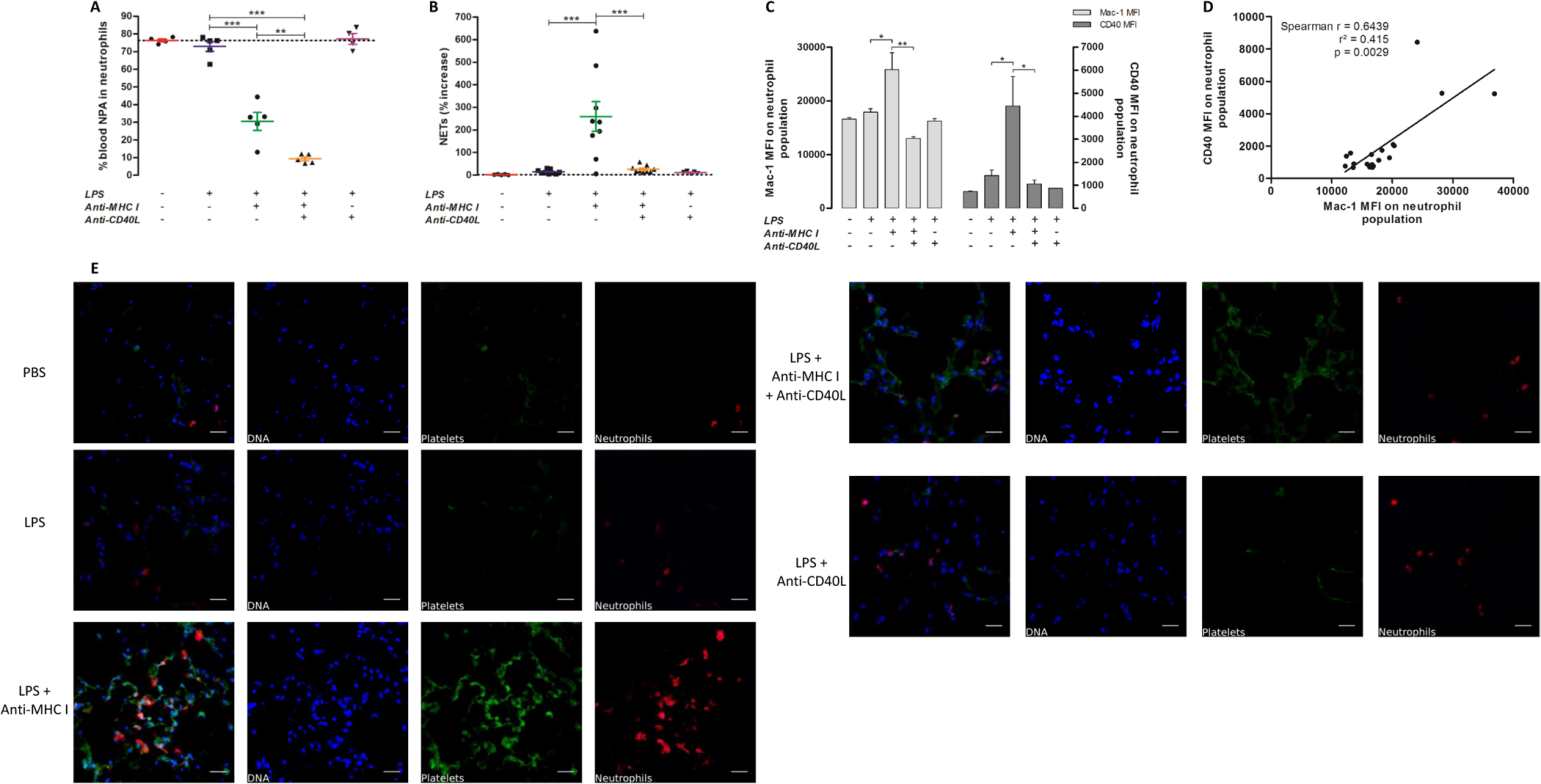
Evaluation of neutrophil and platelet interaction. The proportion of blood NPA in neutrophil populations (**A**) was determined by flow cytometric analysis for each group of mice. The percent NET increase in blood (**B**) was evaluated by immunoassay for each mouse group. Mac-1 and CD40 MFI values for circulating neutrophils (**C** - n = 10) are presented for each mouse group. Data are presented as means ± SEM. *p < 0.05, **p < 0.01, and ***p < 0.001 indicate differences between the [LPS] (n = 4) and [LPS + anti-MHC I] (n = 6) groups; and ^#^p < 0.05, ^##^p < 0.01, and ^###^p < 0^.^001 indicate differences between the [LPS + anti-MHC I] (n = 6) and [LPS + anti-MHC I + anti-CD40L] (n = 6) groups. Correlation between Mac-1 and CD40 expression (**D** - n = 19) was tested using data from all mice. Data are presented as MFI values for each mouse. Spearman’s correlation and the coefficient of determination are indicated by r and r², respectively, and p < 0.05 was considered statistically significant. Immunofluorescence was used to evaluate co-localization of neutrophils and platelets in the pulmonary interstitium (**E**) for each group of mice. DAPI (blue, laser exposition 100 ms/14 V), Alexa fluor® 488 (green, laser exposition 1 s/31.4 V), and Cy5® (red, laser exposition 30 s/64 V), represent nuclei, CD41 and Ly6G, respectively. Overlays were applied using these fluorescence signals (Original magnification x600). Scale bar = 20 µm.

Levels of Neutrophil-platelet aggregate (NPA) formation were evaluated in this model of TRALI, since their presence is a pathogenic risk factor for both sepsis and TRALI^33^. After LPS injection, levels of NPA relative to the neutrophil population were 3%. An injection of anti-MHC I [LPS + anti-MHC I] and anti-CD40L [LPS + anti-MHC I + anti-CD40L] antibodies led to significant decreases (p < 0.001), of 35% to 11%, respectively (Fig. 4A). Furthermore, NPA level was significantly reduced in neutralizing anti-CD40L treated mice compared to anti-MHC I induced TRALI mice (p < 0.01) (Figs 4A and S3A). Neutrophil extracellular traps (NETs), comprised of platelets and neutrophils^34^, were also evaluated. NETosis is particularly increased in the presence of gram-negative bacteria or LPS^35^. NETs were considerably reduced in blood from [LPS + anti-MHC I + anti-CD40L] compared with [LPS + anti-MHC I] mice (Fig. 4B). Moreover, levels of neutrophil surface macrophage-1 antigen (Mac-1) and CD40 were examined following treatment with anti-CD40L antibody. The mean fluorescence intensity (MFI) for Mac-1 differed considerably among groups and was 1.4 and 2 times higher in [LPS + anti-MHC I] mice compared with [LPS] and [LPS + anti-MHC I + antiCD40L] mice, respectively. Similarly, levels of CD40 were significantly higher in [LPS + anti-MHC I] mice (p < 0.05) (Figs 4C and S3B,C). A positive and significant correlation was observed between Mac-1 and CD40 expression (Pearson r = 0.7463 and p = 0.0002; Fig. 4D). Immunofluorescence detection of platelets (CD41) and neutrophils (Ly6G) indicated higher levels of their co-localization in the interstitium in [LPS + anti-MHC I] compared with [LPS] and [LPS + anti-MHC I + anti-CD40L] mice (Fig. 4E). These observations were consistent with the observed reduction in platelet and neutrophil infiltration following treatment with anti-CD40L antibodies (Fig. 2D,E).

### The pulmonary inflammation created by LPS and anti-MHC I injection resolves after 48 h, following parenteral injection of anti-CD40L

**Figure 5:**
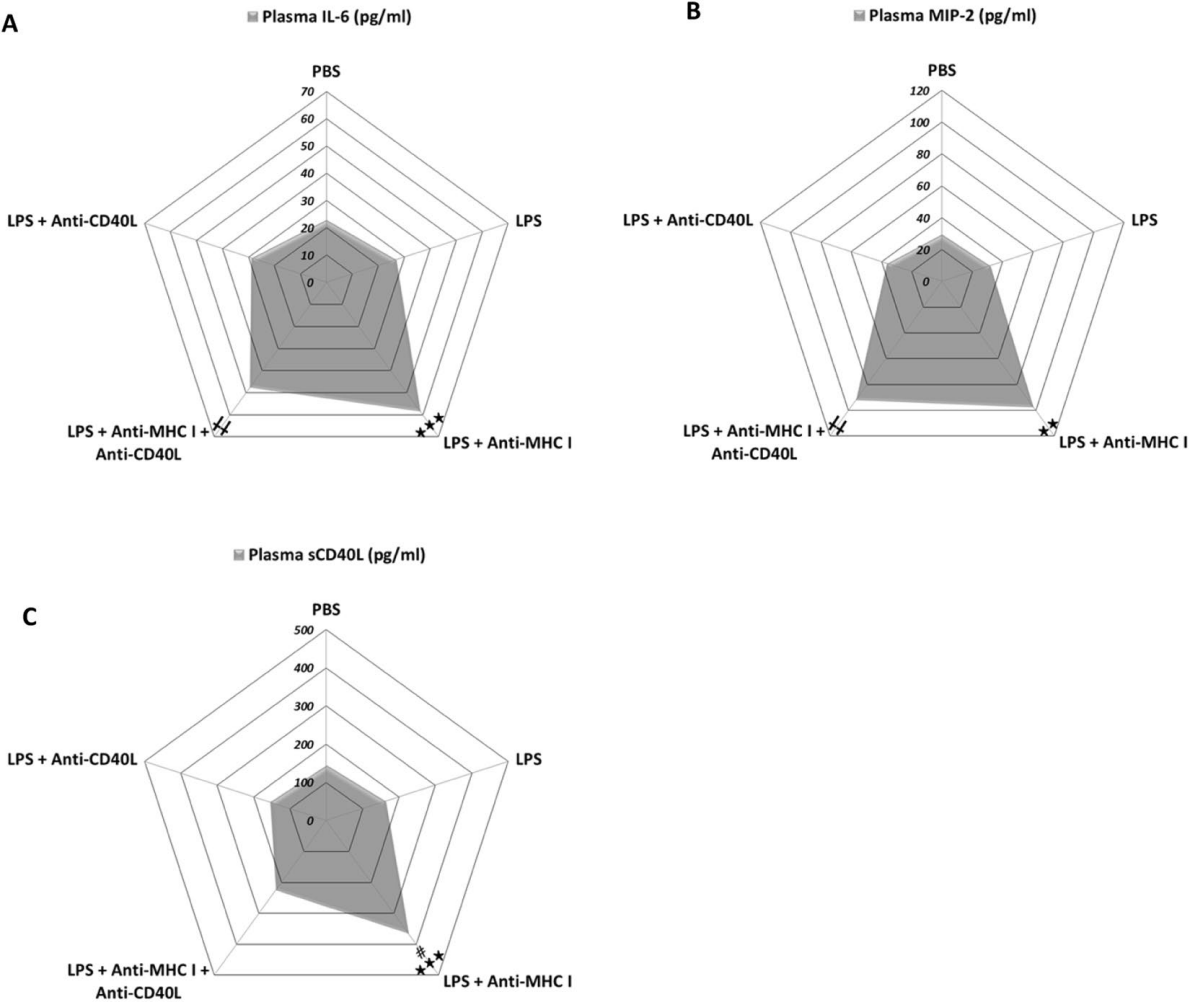
Evaluation of inflammation 2 h after treatment. Levels of IL-6 (**A** - n = 8), MIP-2 (**B** - n = 8), and sCD40L (**C** - n = 8) in plasma were evaluated by immunoassay for each group of mice. Data are presented as means (n = 8). *p < 0.05, **p < 0.01, and ***p < 0.001 indicate differences between the [LPS] and [LPS + anti-MHC I] groups; ^†^p < 0.05, ^††^p < 0.01, and ^†††^p < 0^.^001 indicate differences between the [LPS] and [LPS + anti-MHC I + anti-CD40L] groups; and ^#^p < 0.05, ^##^p < 0.01, and ^###^p < 0.001 indicate differences between the [LPS + anti-MHC I] and [LPS + anti-MHC I + anti-CD40L] groups.

**Figure 6:**
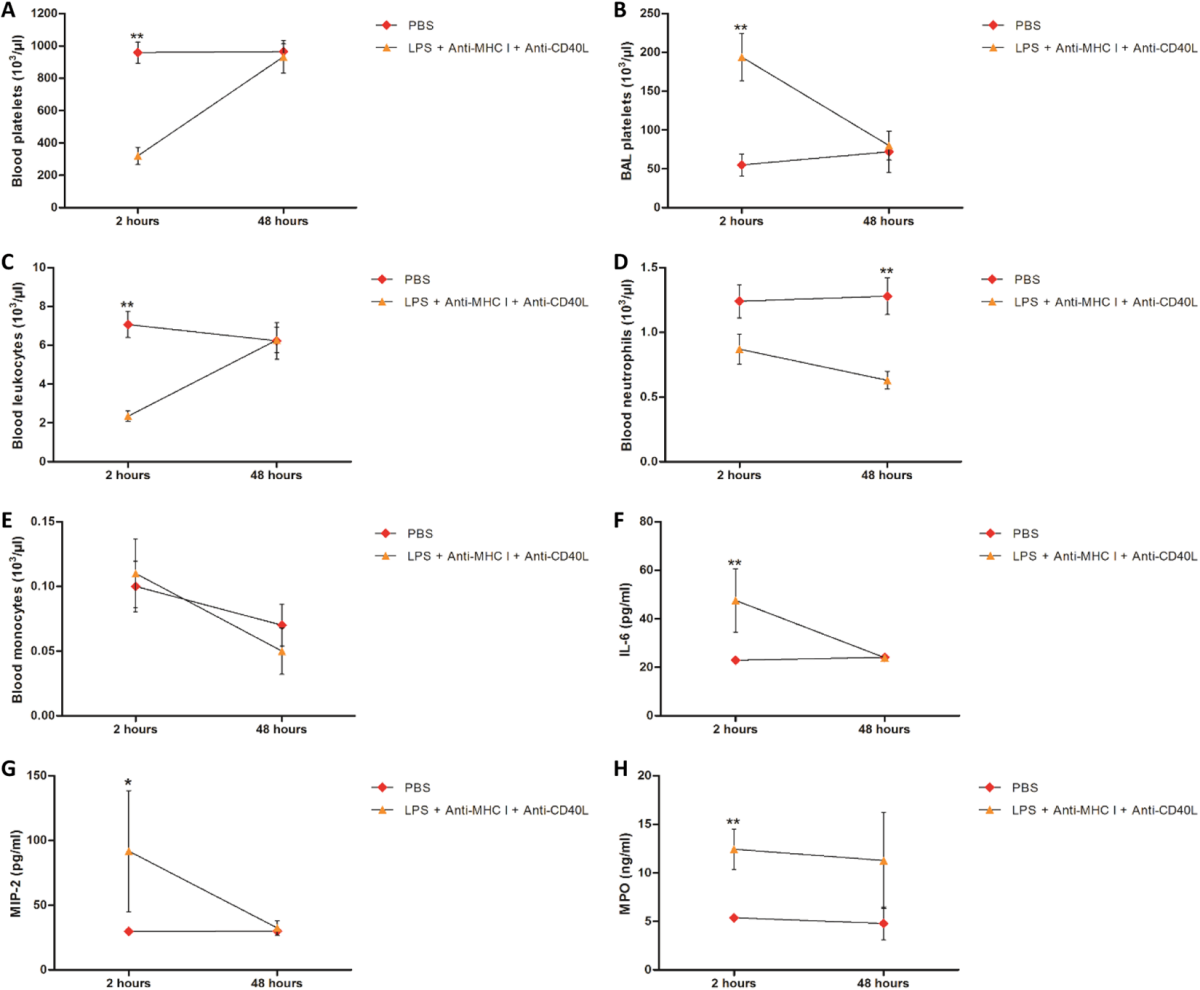
Changes in mouse status 48 h after treatment. Blood (**A** - n = 6) and BAL (**B** - n = 6) platelets, blood leukocytes (**C** - n = 6), neutrophils (**D** - n = 6), and monocytes (**E** - n = 6) were compared between [PBS] and [LPS + anti-MHC I + anti-CD40L] groups at 2 h and evaluated in other [PBS] and [LPS + anti-MHC I + anti-CD40L] mice after 48 h. Plasma IL-6 (**F**), MIP-2 (**G**), and MPO (**H**) were compared between [PBS] and [LPS + anti-MHC I + anti-CD40L] mice after 2 h and evaluated in other [PBS] and [LPS + anti-MHC I + anti-CD40L] mice after 48 h by immunoassay. Data are presented as means. *p < 0.05, **p < 0.01, and ***p < 0.001.

In this model of TRALI, local/regional inflammation was evaluated by measurement of IL-6 and macrophage inflammatory protein 2 (MIP-2) in plasma from the different groups of mice. Both IL-6 and MIP-2 increased by approximately 2- and 3-fold, respectively, compared with controls after LPS and anti-MHC I administration and injection of anti-CD40L did not influence their levels (Fig. 5A,B; Table 1). sCD40L levels in plasma were altered by injection of anti-CD40L in [LPS + anti-MHC I] mice (Fig. 5C; Table 1). Inflammation was evaluated 48 h after challenge with anti-MHC I in [LPS + anti-MHC I + anti-CD40L] mice, in comparison with mice injected with PBS. The aim was to evaluate whether recovery could be considered complete, i.e. equivalent to the basal levels measured in mice that have never undergone an inflammatory stimulus. [LPS + anti-MHC I] mice were not included in this experiment because they were too affected and sacrificed in agreement with the ethic committee (approval number: CU14N11). At 48 h follow-up, [LPS + anti-MHC I + anti-CD40L] mice all survived (Fig. S4), whereas only 40% of [LPS + anti-MHCI] mice survived after 2 h (Fig. 1A). In [LPS + anti-MHC I + anti-CD40L] mice, platelet (Fig. 6A), total leukocyte (Fig. 6C), and monocyte (Fig. 6E) counts returned to baseline after 48 h in blood, as did the platelet count in BAL (Fig. 6B). The difference in neutrophil counts persisted, with significantly fewer circulating neutrophils relative to [PBS] mice (p < 0.01) (Fig. 6D). Using these same mice, we re-evaluated levels of IL-6, MIP-2, and MPO. Despite a two-fold increase in MPO levels in [LPS + anti-MHC I + anti-CD40L] compared with [PBS] mice at 48 h, the increases in these three cytokines tended to recede after 2 h (Fig. 6F–H). In conclusion, in the short term anti-CD40L antibody did not appear to resolve the inflammation induced by anti-MHC I antibody; however, 48 h after anti-MHC I antibody injection, inhibition of CD40/CD40L interaction formation appeared to almost completely resolve the consequences of anti-MHC I antibody administration.

**Table 1:** Inflammatory cytokine levels in treated and untreated TRALI mouse models and controls.

Experimental group	Plasma IL-6 (pg/ml)	Plasma MIP-2 (pg/ml)	Plasma sCD40L (pg/ml)
PBS	22.92 ± 0.38	29.64 ± 0.49	144.38 ± 0.72
LPS	26.80 ± 1.72	32.00 ± 1.81	162.95 ± 1.03
LPS + Anti-MHC I	58.26 ± 9.43***	97.25 ± 22.48**	365.03 ± 2.15***^, ###^
LPS + Anti-MHC I + Anti-CD40L	47.58 ± 5.52**	91.75 ± 23.86**	223.75 ± 5.82
LPS + Anti-CD40L	28.71 ± 1.717	36.43 ± 2.59	157.10 ± 1.60

IL-6, MIP-2, and sCD40L levels were determined in plasma by ELISA for each group of mice. Data are presented as means ± SEM (n = 4–10). **p < 0.01 and ***p < 0.001 indicate significant differences compared with the [LPS] group. ^###^p < 0.001 indicates a significant difference compared with the [LPS + anti-MHC I + anti-CD40L] group.

## Discussion

We used a mouse model to evaluate the role of the CD40/CD40L interaction in TRALI pathophysiology. Anti-CD40L reduced pulmonary edema platelet activation in blood and infiltrated tissues, cellular pulmonary relocation and neutrophil activity in the lungs. Anti-CD40L also appeared to limit intercellular communication, particularly between neutrophils and platelets, evidenced by decreases in NPA formation and NETs, which correlated with low Mac-1 expression on neutrophils. The inflammatory response is propagated and matured by a series of biochemical events involving the local vascular system, the immune system, and different cells within the injured tissue. Cytokine/chemokine is a part of inflammation. The main limitation of this treatment was that inflammation was not prevented – globally – following the injection of anti-CD40L. Injecting anti-CD40L reduced the infiltration of leukocytes, and subsequently controlled inflammation tissue damage. The presence of CD40L receptor on neutrophil (CD40 and Mac-1), could reduce circulating sCD40L concentrations, even if we observed a platelet activation phenotype after neutrophils binding. Our data suggest a protective role of neutralizing anti-CD40L mAb to prevent pulmonary edema formation. These findings do not accord with previous observation investigating the influence of CD40/CD40L interaction in a “one-hit” mouse model of TRALI. Treatments with ciglitazone and neutralizing anti-CD40L do not prevent TRALI development in mice^23^. Nevertheless, the used mouse model was different than the one used in our study, considered as the reference mouse model of TRALI^6^. Effectively, the used mouse model consisted to a “one-hit” model of TRALI with a single anti-MHC I injection at 4.5 mg/kg, whereas we used a “two-hit” mouse model more representative of the recognized “two-hit” hypothesis of human TRALI^4^. This difference may explain the absence of protection against the development of TRALI observed in our study during the inhibition of the CD40/CD40L interaction. Tuinman *et al*. investigated the role of CD40L in a murine model of antibody-mediated TRALI as a mediator of lung injury^23^; Mice were challenged with a MHC-1 antibody that induced TRALI, evidenced by pulmonary edema; compared to an isotype antibody infusion, this was accompanied by significantly elevated levels of total proteins and of keratinocyte-derived chemokine bronchoalveolar fluid, and macrophage inflammatory protein-2. In addition, McKenzie CG *et al*. interestingly suggested that peripheral blood monocytes were the source of the MIP-2 and that both were essential for the initiation of TRALI. Depletion of peripheral monocytes significantly reduced levels of MIP-2 and sequestration of pulmonary neutrophils, suggesting that the source of production of MIP-2 and activation of neutrophils is circulating monocytes as opposed to other types of cells such as pulmonary endothelium^36^.

Our data suggest that anti-CD40L mAb inhibits neutrophil migration, rather than activating or regulating inflammation in TRALI in mice. These results are consistent with reports demonstrating the direct impact of antibodies on CD40/CD40L interaction inhibition, via Mac-1 on the neutrophil surface^27,37,38^. Mac-1 is directly involved in neutrophil migration^39^, cellular interactions between neutrophils and platelets via the Mac-1/GPIbα complex^40^, and communication between neutrophils and endothelial cells^38,39^. The interaction between platelets and neutrophils may be responsible for amplified platelet sCD40L production, promoting complex formation^41^. Platelet-secreted sCD40L induces chemokine release by endothelial cells, promoting neutrophil attachment and migration in the alveolar space, conditioned by TRALI^42^. Neutrophil migration from the blood to the lungs may be dependent on platelet activation, since the close communication between these two cell types enhances neutrophil migration^34,43^. Notably, the lungs are a significant source of thrombopoiesis in mice, similar to the bone marrow^44^. The changes in BAL platelet levels observed in this study are consistent with both TRALI-related platelet and neutrophil migration and compensatory pulmonary thrombopoiesis due to TRALI-induced thrombocytopenia. Weissmüller T *et al*. showed that comigration of platelet-PMNs was observed in intestinal tissue derived from inflammatory bowel disease patients. The translocated platelets were found to release large amounts of ATP metabolized to adenosine through a 2-step enzymatic reaction mediated by ecto-nucleotidases, including CD73 and ecto-nucleoside triphosphate diphosphohydrolases (ecto-NTPDases), expressed on the apical membrane of the intestinal epithelial cells^45^. We could not exclude that anti-CD40L mAbs could activated platelets, as described by Langer F. *et al*.^46^. These authors demonstrated that platelets can be *in vitro* activated by immune complexes (IC) consisting of anti-CD40L mAbs and CD40L (anti-CD40L IC) and that this activation is dependent on the IgG Fc receptor on the platelet surface, FcγRIIa. However, in our model, we do not observe plasma PF4 concentration as a platelet activated maker, comparing [LPS + anti-CD40L] *vs* [LPS]. A similar comment was observed concerning the MPV. Inflammatory activation is effectively potentiated by a very short LPS priming. We suggest that in signalling immune or non-immune cells through TLR4, LPS triggers either post-translation modifications or subcellular protein relocation required for full inflammatory activation. LPS may also acutely regulate the expression and/or functions of yet-undefined accessory proteins required for optimal inflammatory activation.

The main limitations of this study are: (i) applications and limitations of mouse models for understanding Human pathology and particularly inflammatory pathophysiology; (ii) lack of direct visualization of platelet and neutrophil relocation from the vasculature to the lung parenchyma; (iii) lack of direct measurement of communication between neutrophils and endothelial cells, a key parameter in the extravasation of neutrophils to the alveolar space^39^; (iv) lack of distinction between platelets recruited from the periphery and those produced locally, in the lungs; v) we focused on CD40 and/or CD40L in this study, aware that other soluble factors (Biologic response modifiers, Bioactive lipids…) play an additional role concerning the induction of TRALI and vi) base on “resource equation” method”^47^, we noted that our “E” value is more than 20 which is the acceptable limit and hence can be considered as more than sufficient sample size. The use of neutralizing antibodies and platelet targeting antagonist/agonist BRMs should be a pathway to prevent the development of pulmonary edema. Several mouse models were used to focus on the different steps of TRALI and ALI caused by direct injury as well as stimuli for acid, LPS or mechanical ventilation^48^. Nonetheless, many pathogenesis details, particularly with regard to non-immune mediated TRALI, are poorly understood and different animal models have been established for better investigation. Our study—similarly to most studies by others—investigated the pathogenesis of TRALI; however, none does well mimic the clinical situation of trauma, surgery and intraoperative blood loss, as most studies ignore that patients with trauma and hemorrhage are more susceptible to development of TRALI. Moreover, in a recent study Beura L.K *et al*.^49^ highlighted the environmental effects of basal immune and infection response and suggested that restoring physiological microbial exposure in laboratory mice could provide a relevant tool for modeling immunological events in free-living organisms, including humans. Finally, interestingly, Kapur R *et al*. detailed that mice with severe combined immunodeficiency could be considered to investigation of the pathogenesis of antibody-mediated TRALI^50–52^. Elegantly, using a C57BL/6 – IL10 KO mice, the author demonstrated, that recipient resistance to the effects of TRALI-inducing antibodies is mediated through the IL-10 transfusion-related Treg – DC axis.

Our study did not explore monocytes in depth, only their numbers; although a previous study demonstrated that 34-1-2 s anti-MHC I antibody can directly activate this cell type^36^. ALI, and TRALI in particular, likely involves various cell types in addition to neutrophils. Pulmonary edema is an under-recognized and potentially serious complication during or soon after blood transfusion. Our study shows a dramatic deterioration of the lungs, a significant increase of a wet lung mass/body mass ratio and total protein in bronchoalveolar lavage (BAL) in [LPS + anti-MHC I] mice compared with [LPS] controls. Moreover, H&E staining of the pulmonary interstitium revealed pulmonary infiltration, evidenced by pulmonary cellular exudate, in [LPS + anti-MHC I] mice, while the pulmonary parenchyma of [LPS] controls and [LPS + anti-MHC I + anti-CD40L] treated mice showed no evidence of infiltration. All these parameters could be considered as an edema characteristic of TRALI. However, we could not exclude an acute hemodynamics effects combined with lung injury.

Our study shows a regulation of neutrophil and platelet activity under neutralizing anti-CD40L mAb, but this treatment may also prevent other inflammatory cell activity participating in TRALI development. Indeed, several studies mentioned other cells, with the capacity to expose CD40 and/or CD40L on their surface, implicated in some experimental TRALI pathophysiology, like dendritic cells^50^, T regulatory lymphocytes^50^, pulmonary macrophages^53^ and monocytes^36^.

## Conclusions

Overall, our experiments used a mouse as a model for TRALI investigation; data indicate that improvement of the conditions under which platelet concentrates are prepared and stored to reduce sCD40L levels are central to minimize the risk of TRALI. We anticipate that better understanding of patient risk factors and the first hit of TRALI could suggest prophylactic and curative treatments. Identification of patients at risk for TRALI will enable preemptive personalized treatments, facilitating both improved patient management and cost reduction.

## Materials and Methods

### Mice

Male BALB/c wild-type mice (8–12 weeks old) were purchased from Charles River (Charles River, Wilmington, USA). Animals handled carefully/deftly to minimize stress before the experiments with i) 4 Adult Mice Per Cage, ii) the animals were randomly housed before the experiment, iii) animals were selected at random for outcome assessment, iv) limitation of the study described in the discussion section, v) Mice were allowed 1 week of acclimation before experimental manipulation was initiated and vi) during acclimation, mice had free access to water bottles with reverse-osmosis water. All experiments were conducted in accordance with institutional guidelines and approved by the University of Saint-Etienne. Experiments used ≥4 mice per group. Experimental groups were: PBS [baseline]; LPS [control]; LPS + Anti-MHC I mAb; LPS + Anti-MHC I mAb + neutralizing anti-CD40L mAb [treated mice]; and LPS + Anti-CD40L mAb [treatment control].

### Animal experiments

Male H2K^d^ BALB/c mice were primed by intraperitoneal (*i.p*.) injection of LPS (0.1 mg/kg, extracted from *Escherichia Coli* 0111; InvivoGen, San Diego, USA), 24 h before challenge with intravenous (*i.v*.) anti-MHC I monoclonal antibody (mAb 34-1-2 s; 1 mg/kg; H2K^d^; IgG2a, κ) or IgG2a, κ isotype control (eBM2a) (eBioscience, San Diego, USA). Mice in the treatment group were i.v administered with 4 mg/kg anti-CD40L mAb (MR1) or IgG3, κ Isotype control (E36-239) (BD Pharmingen, Franklin Lakes, USA) 30 min prior to challenge with anti-MHC I mAb or isotype control (Fig. S1). Ketamine (100 mg/kg) and xylazine (10 mg/kg) were administered intraperitoneally when mice appeared moribund or after 2 h. After death, mouse lungs were collected and placed in 4% paraformaldehyde (Sigma Aldrich, Saint-Louis, USA) overnight.

### TRALI development and edema evaluation

Mouse temperatures were measured using a rectal probe and digital thermometer (Bioseb, Pinellas Park, USA), prior to anti-MHC I injection and then every 10 min for 2 h, or until death. Mouse survival rates were evaluated for 2 h and at 48 h following treatment. First set of mice were used to calculate wet lung to body weight ratio. Other set of mice were used to obtain bronchial lavage fluid (BAL) through a tracheotomy with a 25-gauge catheter before the pneumonectomy. Lavage was conducted using a 1 ml injection of cold PBS flushed back three times. BAL cells were centrifuged at 491 g for 10 min and resuspended in PBS. BAL platelets were enumerated using an MS4™ Hematology Analyzer (Melet Schloesing Laboratoires, Osny, France). BAL total proteins were evaluated by Bradford assay. Peripheral-blood cell number and MPV were determined using the MS4™. Intracardiac puncture was conducted with a 25-gauge needle and anticoagulant citrate-dextrose solution (Sigma Aldrich, Saint-Louis, USA). Tail vein injections used a 30-gauge needle. Plasma samples were collected and stored. The BAL platelet ratio was calculated as follows:

$$(({{\rm{BAL}}}_{{platelets}}/{{\rm{BAL}}}_{{platelets}}+{{\rm{Blood}}}_{{platelets}}))\times 100$$

### Lung sections

Lungs, for which no BAL has been performed, were embedded in OCT compound (CML, Nemours, France), placed over liquid nitrogen to induce rapid solidification. For hematoxylin and eosin (H&E) staining (Sigma-Aldrich, Saint Louis, USA), immunohistochemistry, and immunofluorescence, 8 µm sections were prepared using a cryostat microtome (Leica Microsystem, Nanterre, France).

### H&E staining and immunohistochemistry

Pulmonary cellular exudate area was evaluated through stereological assessment, as previously described^54^, after H&E staining. For immunohistochemistry, endogenous peroxidase activity was blocked with 0.3% H_2_O_2_. Bovine serum albumin (BSA, 3%) (Sigma-Aldrich, Saint Louis, USA) was used to block nonspecific hybridization, sections were incubated for 1 h with anti-CD41 mAb (MWReg30, 2.5 µg/ml) or anti-Ly6G mAb (1A8, 5 µg/ml; BD Biosciences, Franklin Lakes, USA). Sections were then incubated with biotinylated secondary anti-rat mAb (G15-337, 5 µg/ml; BD Biosciences, Franklin Lakes, USA) for 30 min, followed by pre-diluted streptavidin-Horseradish peroxidase and 3, 3′-Diaminobenzidine. Sections were treated by successive immersion in hematoxylin, alcohol (95%, 100%) and xylene (Sigma-Aldrich, Saint Louis, USA). Observations were made with a Nikon Eclipse Ti-S microscope, Nikon DS-RI2 camera and Nikon NIS-Elements software (Nikon, Champigny sur Marne, France). Images were processed with Image J software^55^.

### Immunoassays

MPO and PF4 (Mouse Myeloperoxidase DuoSet ELISA and Mouse CXCL4/PF4 DuoSet ELISA, R&D Systems, Minneapolis, USA) were measured in plasma and BAL according to the manufacturer’s instructions. Plasma IL-6, MIP-2 (Mouse IL-6 DuoSet ELISA and Mouse CXCL2/MIP-2 DuoSet ELISA R&D Systems, Minneapolis, USA), and sCD40L (Mouse TH17 Magnetic Bead Panel Multiplex Assay, Merck Millipore, Billerica, USA) were measured according to manufacturer’s instructions. NETs were evaluated in mouse plasma by ELISA as previously described^34^. Values for soluble NET formation are expressed as percentage increase in absorbance above control, calculated as follows:

$$(({{\rm{OD}}}_{{x}}-{{\rm{OD}}}_{blank})/{{\rm{OD}}}_{blank}))\times 100$$

### Neutrophil-platelet aggregate (NPA) assay

Immediately following whole blood collection, flow cytometry was used to evaluate NPA formation. PE-anti-CD45 mAb (30-F11) and APC-anti-Ly6G mAb (1A8) (BD Biosciences, Franklin Lakes, USA) were used to identify neutrophils. FITC-anti-CD41 mAb (MWReg30) was used to identify platelets among the CD45/Ly6G neutrophil population; a positive FITC signal in this population was considered to indicate NPA. Analysis was performed using FACSCANTO II (BD Biosciences, Franklin Lakes, USA) and FlowJo™ software (FlowJo, Ashland, USA).

### Phenotypic characterization of neutrophils

Neutrophils were identified in whole blood using PE-anti-CD45 mAb (30-F11) and APC-anti-Ly-6G mAb (1A8). Neutrophil surface Mac-1 (CD18) and CD40 were identified with FITC-anti-CD18 mAb (M18/2) and Pacific Blue^TM^-anti-CD40 mAb (3/23) (BioLegend, San Diego, USA). Flow cytometry and software analysis were as described above.

### Immunofluorescence assays

Pulmonary-interstitial co-localization of platelets (CD41) and neutrophils (Ly6G) was by indirect immunofluorescence. Pulmonary sections were blocked with 10% BSA and incubated with anti-CD41 mAb (MWReg30, 1 µg/ml; Abcam, Cambridge, USA) and anti-Ly6G mAb (1A8, 5 µg/ml) simultaneously for 1 h in a humidified chamber. Next, secondary Alexa fluor™ 488-anti-rabbit mAb (2 µg/ml) and Cy5™-anti-rat mAb (5 µg/ml; Abcam, Cambridge, USA) were added for 1 h. Nuclei were stained with 4’, 6-diamidino-2-phenylindole (DAPI). Images and overlay were treated with Image J software^55^.

### Statistical analyses

Statistical analyses were performed using GraphPad Prism 5 software (Graph ad, San Diego, USA). Unpaired Student’s t-tests were used for comparisons between two groups if the data were normally distributed (as determined by Kolmogorov-Smirnov test), with Mann Whitney tests applied for non-normally distributed data. ANOVA with post-hoc Bonferroni correction was used for comparisons among more than two groups of data with normal distribution, with the Kruskal-Wallis and Dunn’s post-hoc tests applied for comparisons among non-normally distributed data. Temperature and mouse TRALI scores over 48 h were calculated using two-way analysis of variance. Correlations were by Spearman’s test. P-values < 0.05 were considered significant.

### Study approval

Animal use was by the authorization of the Ethics Committee of the French Ministry of Higher Education and Research (approval number: CU14N11) and all experiments were performed in accordance with relevant guidelines and regulations.

## Supplementary information

Supplementary Dataset 1
