# Supplementary material for: Evidence of CD40L/CD40 pathway involvement in experimental transfusion-related acute lung injury
Source: Sci Rep. 2019 Aug 29;9:12536. doi: 10.1038/s41598-019-49040-0 (PMC6715651; doi:10.1038/s41598-019-49040-0)
Supplement: Supplementary file 1 — Supplementary Dataset 1 [file 41598_2019_49040_MOESM1_ESM.docx]

**Evidence of CD40L/CD40 pathway involvement in experimental transfusion-related acute lung injury**

**Running Title :** Role for CD40/CD40L in immune TRALI

Sofiane Tariket,^1,2^ Hind Hamzeh-Cognasse,^1^ Sandrine Laradi,^1,2^ Charles-Antoine Arthaud,^2^ Marie-Ange Eyraud, ^2^ Thomas Bourlet, ^1^ Philippe Berthelot, ^1^ Olivier Garraud,^1,3^ and Fabrice Cognasse^1,2,*^

^1^Université de Lyon, GIMAP-EA3064, Saint-Etienne, France; ^2^Établissement Français du Sang Auvergne-Rhône-Alpes, Saint-Etienne, France; ^3^Institut National de la Transfusion Sanguine, Paris, France

**Supplemental data**


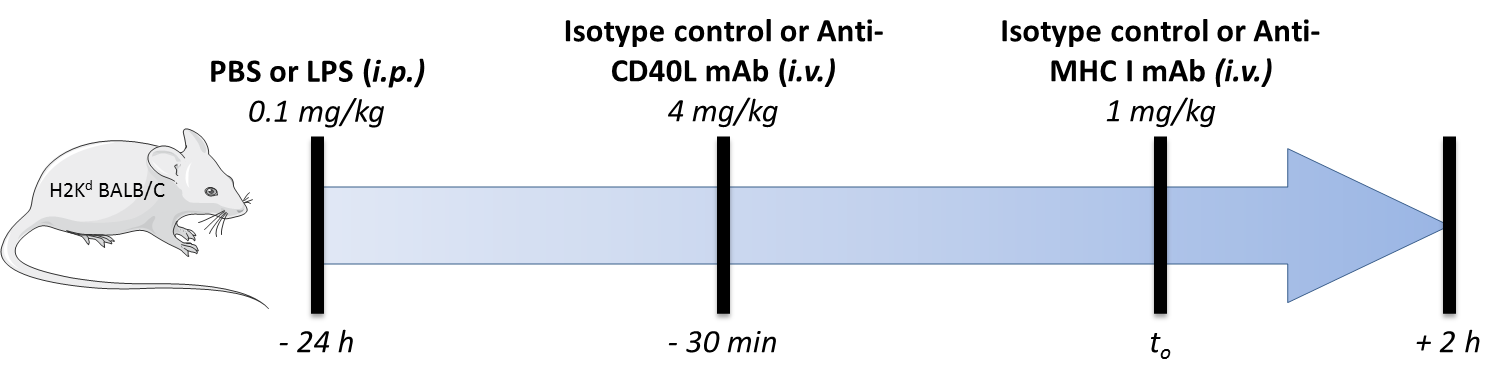


**Figure S1: TRALI experimentation**

**Description of data:** The different injections performed during the TRALI mouse study are presented. PBS or LPS (at 0.1 mg/kg) was injected, intraperitoneally, 24 hours before isotype control or anti-MHC I mAb (at 1 mg/kg) intravenous injection. The injection of isotype control or neutralizing anti-CD40L mAb (at 4 mg/kg) was performed, intravenously, 30 minutes prior to isotype control or anti-MHC I mAb intravenous administration. I confirm that this image was drawn by one of co-authors (Sofiane TARIKET).


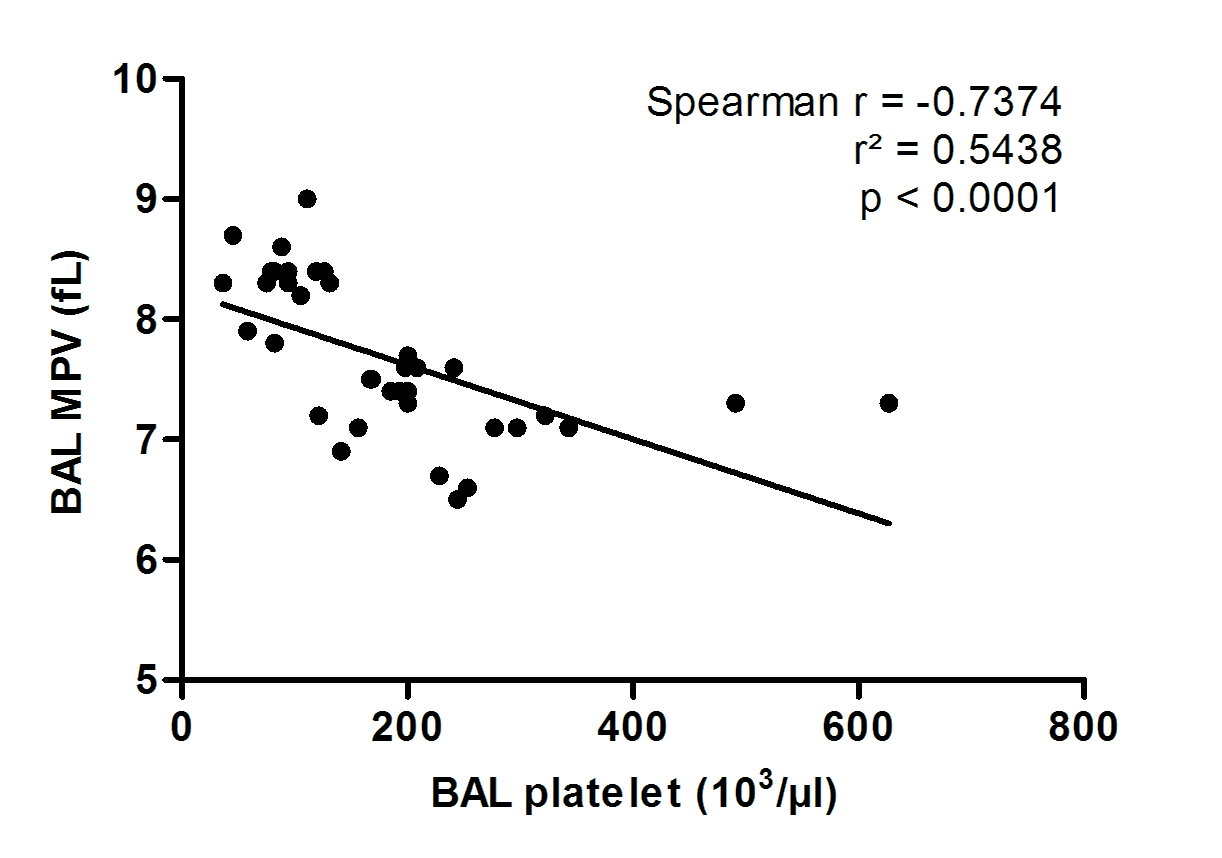


**B**

**A**


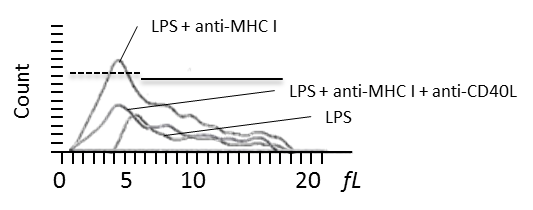


Figure S2: BAL MPV evaluation

**Description of data:** Correlation was tested between BAL platelet count and BAL MPV, including all mice (n = 38). Spearman’s correlation and the coefficient of determination are represented respectively by r and r² symbols and p < 0.05 is considered statistically significant. BAL platelet count and BAL MPV are significantly and negatively correlated (p < 0.0001 and r = -0.7374) (**A**). An overlay of a BAL [LPS], [LPS + anti-MHC I] and [LPS + anti-MHC I + anti-CD40L] mouse MS4® platelet histogram is represented. We hypothesize that the dotted line represents *de novo* platelets and the full line is characteristic of migrated and activated platelets. In this histogram, [LPS] mouse MPV is 8.4 *fL*, [LPS + anti-MHC I] mouse MPV is 6.7 *fL* and [LPS + anti-MHC I + anti-CD40L] mouse MPV is 7.5 *fL* (**B**). These results show that *de novo* platelets from the lungs presumably compensate for peripheral blood thrombocytopenia, particularly in the [LPS + anti-MHC I] mice and to a lesser extent, due to the limited but persistent thrombocytopenia, in the [LPS + anti-MHC I + anti-CD40L] mice.

**A**


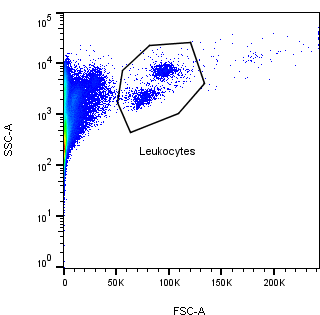

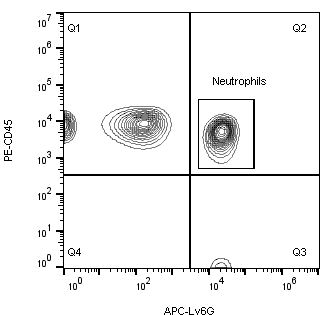

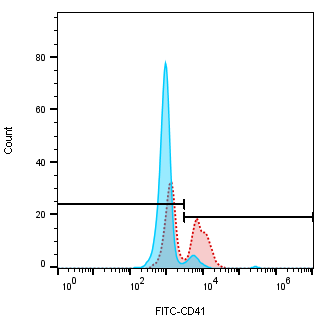


**B**


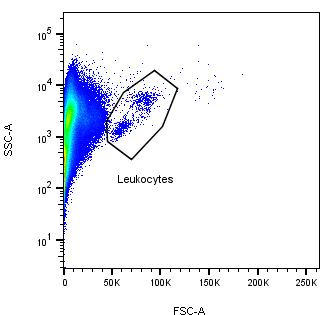

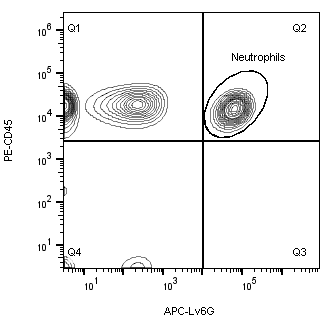

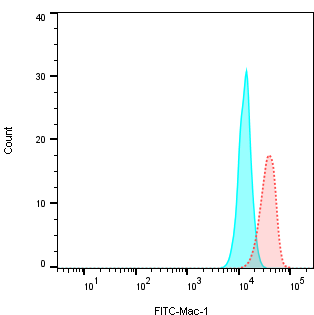


**C**


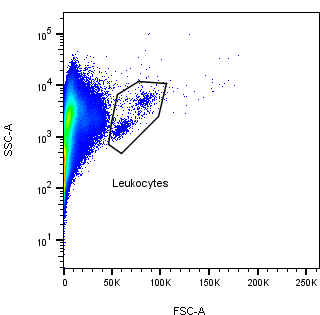

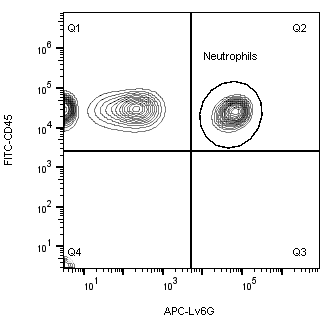

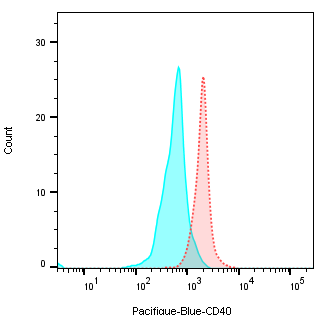
 **Figure S3: NPA, Mac-1 and CD40 cytometry process**

**Description of data**: The cytometry process is presented. NPA frequency was evaluated directly in the CD45+/Ly6G+ neutrophil population via a FITC-anti-CD41 mAb fluorescence signal (**A**). Mac-1 MFI was evaluated directly in the CD45+/Ly6G+ neutrophil population via a FITC-anti-Mac-1 mAb fluorescence signal (**B**). CD40 MFI was evaluated directly in the CD45+/Ly6G+ neutrophil population via a Pacific Blue^TM^-anti-CD40 mAb fluorescence signal (**C**). The red spectrum represented a [LPS + anti-MHC I] mouse and the blue spectrum represents a [LPS + anti-MHC I + anti-CD40L] mouse.


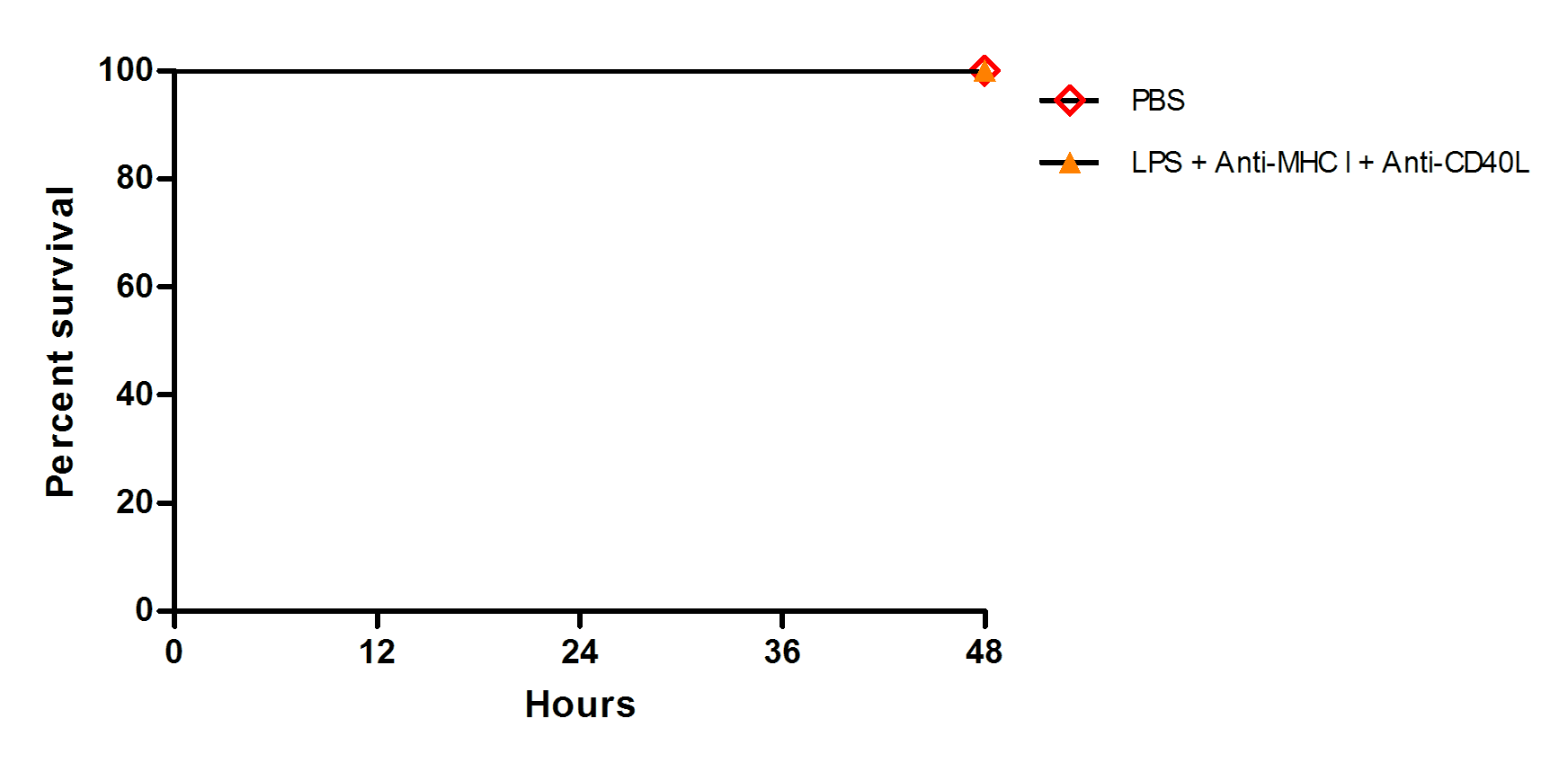


Figure S4: Survival rate after 48 hours

**Description of data:** The mortality percentage is represented for the [PBS] and [LPS + anti-MHC I + anti-CD40L] groups over 48 hours (n = 5). No survival difference was observed between these two groups after 48 hours.
